# Ultrasound-guided single popliteal sciatic nerve block is an effective postoperative analgesia strategy for calcaneal fracture: a randomized clinical trial

**DOI:** 10.1186/s12891-021-04619-5

**Published:** 2021-08-27

**Authors:** Yanan Li, Qi Zhang, Ying Wang, Chunping Yin, Junfei Guo, Shiji Qin, Yahui Zhang, Lian Zhu, Zhiyong Hou, Qiujun Wang

**Affiliations:** 1 Department of Anesthesiology, the Third Hospital of Hebei Medical University, NO.139, Ziqiang Road, Shijiazhuang, Hebei Province China; 2 Department of Anesthesiology, Children’s Hospital of Hebei province Affiliated to Hebei Medical University, Shijiazhuang, Hebei China; 3 Department of Orthopaedics, the Third Hospital of Hebei Medical University, Shijiazhuang, Hebei China; 4 Key Laboratory of Orthopaedic Biomechanics of Hebei Province, Shijiazhuang, Hebei China; 5 Department of Foot and Ankle Surgery, the Third Hospital of Hebei Medical University, Shijiazhuang, Hebei China; 6 Department of Nursing, the Third Hospital of Hebei Medical University, Shijiazhuang, Hebei China

**Keywords:** Sciatic nerve block, Calcaneal fracture, Analgesia, Ultrasound, Adverse reactions, Randomized trail

## Abstract

**Objectives:**

The aim of this study was to evaluate the postoperative analgesia effect of ultrasound-guided single popliteal sciatic nerve block for calcaneal fracture.

**Methods:**

A total of 120 patients scheduled for unilateral open reduction and internal fixation of calcaneal fracture were enrolled in this prospective randomized study. Patients in group B received ultrasound-guided single popliteal sciatic nerve block after operation, but Patients in group A did not. All patients received patient-controlled intravenous analgesia (PCIA) after operation. The time to initiation of PCIA, the time of first pressing the analgesia pump, duration of analgesia pump use and the total number of times the patient pressed the analgesia pump were recorded. The time of rescue analgesia and the adverse reactions were recorded. Pain magnitude of the patients immediately after discharge from operating room (T1), and at 4th (T2), 8th (T3), 12th (T4), 16th (T5), 24th (T6) and 48th (T7) h after the operation were assessed with visual analog scale (VAS). In addition, patient, surgeon and nurse satisfaction were recorded.

**Results:**

The VAS scores at T2 ~ T5, the time of rescue analgesia and the adverse reactions, the total number of times the patient pressed the analgesia pump were significantly declined in group B (*p* < 0.001). The time to initiation of PCIA, the time of first pressing the analgesia pump, duration of analgesia pump use were prolonged and patient surgeon and nurse satisfaction were improved in group B (*p* < 0.05).

**Conclusion:**

Ultrasound-guided single popliteal sciatic nerve block is an effective postoperative analgesia strategy for calcaneal fracture.

**Trial registration:**

ChiCTR, ChiCTR2100042340. Registered 19 January 2021, URL of trial registry record: http://www.chictr.org.cn/showproj.aspx?proj=66526.

## Introduction

Calcaneal fracture is a common injury of foot and ankle accompanied by severe and difficult-to-treat pain. For patients with calcaneal fractures, surgery is generally the mainstay of treatment to relieve pain and correct foot dysfunction [1]. However, the surgical treatment of calcaneal fractures can result in significant postoperative pain for at least 24 h [2]. In addition, severe pain often leads to physical inactivity, potentially leading to hematoma, incision dehiscence or infection, thereby increasing the difficulty of postoperative nursing and the workload of medical staff [3, 4]. Therefore, effective postoperative analgesia is of great significance to relieve patients’ pain and promote postoperative recovery.

Traditionally, during the surgical treatment of calcaneal fracture, spinal anesthesia combined with postoperative analgesia pump are usually chosen by many anesthesiologists [5, 6]. But a few hours after analgesic surgery, the analgesic effect of spinal anesthesia is gradually decreased, and patients will experience unbearable pain [7] . Accordingly, the patient will repeatedly press the pump, or ask the doctor to administer extra analgesic drugs. In a short period of time, a large number of analgesics are injected into the patient’s body, which is more likely to cause adverse reactions such as nausea, vomiting, shivering, respiratory depression and skin itching [8–10]. In addition, the analgesic effect of intravenous analgesics is far from perfect, which may bring painful postoperative experience to patients and cause certain psychological trauma [11].

Due to various advantages, such as better comfort and safety, higher patient satisfaction, shorter hospitalization and less hospitalization cost, ultrasound-guided peripheral nerve blocks (PNB) has gained increasing popularity among anesthesiologists and patients [12–14]. Both prospective or retrospective studies and meta-analysis have consistently confirmed that peripheral nerve block has fewer unwanted effects on cardiopulmonary function, as well as significantly fewer related complications, as compared with other anesthesia methods [15–17]. Because of the particularity of the sciatic nerve, anesthesiologists can perform sciatic nerve block at many parts of the body surface, such as superior sciatic block, popliteal block and ankle joint block. Popliteal or ankle blocks are used most frequently in foot and ankle surgery. A large number of studies have confirmed that the popliteal approach sciatic nerve block can be safely and effectively applied to patients undergoing foot and ankle surgery, significantly reducing the pain of patients during the operation. In addition, the analgesic effect can last approximately 15 h after operation, effectively reducing the dosage of opioid analgesics as well as a series of complications caused by the application of opioid drugs [18].

In recent years, some clinicians have carried out a series of explorations on the application of peripheral nerve tissue in postoperative analgesia, and obtained some exciting results [12]. However, few data are currently available in the literature evaluating analgesic effect of sciatic block for patients receiving operative treatment of calcaneal fracture. To obtain more information on this topic, we conducted this prospective randomized study to test the hypothesis that, as a postoperative analgesia program for calcaneal fracture, ultrasound-guided single popliteal sciatic nerve block could provide a better analgesic effect, reduce the side effects of analgesic drugs, and promote the process of postoperative recovery. The results of this study will provide a novel analgesic method for patients receiving operation of calcaneal fracture.

## Methods

### Ethics

This randomized clinical study was approved by Ethics Committee of the Third hospital of Hebei Medical University (No: 2019–022-1) and registered on Chinese clinical trail registry (ChiCTR2100042340). The study was performed in accordance with the Declaration of Helsinki. All patients signed informed consent before operation.

### Patients population

Patients aged ≥18 years with ASA physical status I ~ II and Sanders type II or III who were scheduled for unilateral open reduction and internal fixation (ORIF) of calcaneal fracture from 20th January 2021 to 15th April 2021 in department of Foot and Ankle Surgery were included in this study. A calcaneus plate (Wuhan Tianying Medical Equipment Co., Ltd) was used for internal fixation for all patients. Exclusion criteria included body mass index ≥35 kg/m^2^, medical contraindication to regional nerve block (eg, allergy, bleeding disorders, localized infection and neurologic disease) and to spinal anesthesia (eg, bleeding disorders, localized infection, receiving lumbar surgery and central nervous system disease); the injury that lasted more than 5 days; patients with wound infection; pregnant patients; patients receiving chronic pain treatment, having drug abuse history and unable to use intravenous analgesia pump independently, and having respiratory or cardiac disease. Patients who switched to general anesthesia due to unsuccessful spinal anesthesia would be excluded from this study.

### Randomization and blinding

All patients were divided into two groups: group A (not receiving postoperative sciatic nerve block) and group B (receiving postoperative sciatic nerve block) by random number scale. All patients were blinded to the group allocations. The anesthesiologist who assessed the patients and the person who analyzed the data were blinded to the group allocation.

### Anesthesia application

After entering the operating room, the peripheral vein of upper limb was opened and 500 mL sodium acetate Ringer solution was intravenously injected within 30–40 min. All patients received routine intraoperative monitoring, such as oxygen saturation (SpO2), electrocardiography (ECG), and non-invasive blood pressure (NBP) measurements. All patients were placed in lateral decubitus position. The skin site was aseptically prepared and draped for operation. Subsequently, 3–5 ml of 1% lidocaine (100 mg/5 ml, Batch number: A20H030, Hebei Huachen Pharmaceutical Co., Ltd) was injected into the skin, subcutaneous tissue and the interspinous ligament in L4-L5 or L3-L4 intervertebral space. The procedure was performed using a 25G Quincke spinal needle (AS-E/S II, Batch number: 1909A1001, Zhejiang Sujia medical device Co., Ltd). After passing arachnoid membrane and entering the subarachnoid space, the plunger was gently withdrawn and there was free flow of the spinal fluid. After the cerebrospinal fluid was withdrawn smoothly, 3 ml of 0.5% bupivacaine (25 mg/5 ml, Batch number: D19G19, Shanghai Zhaohui Pharmaceutical Co., Ltd) was injected at a rate of 1 ml/10 s, and the anesthesia level was controlled at T10-T12. Subsequently, all patients received unilateral ORIF of calcaneus.

### Analgesia protocol

**Fig. 1:**
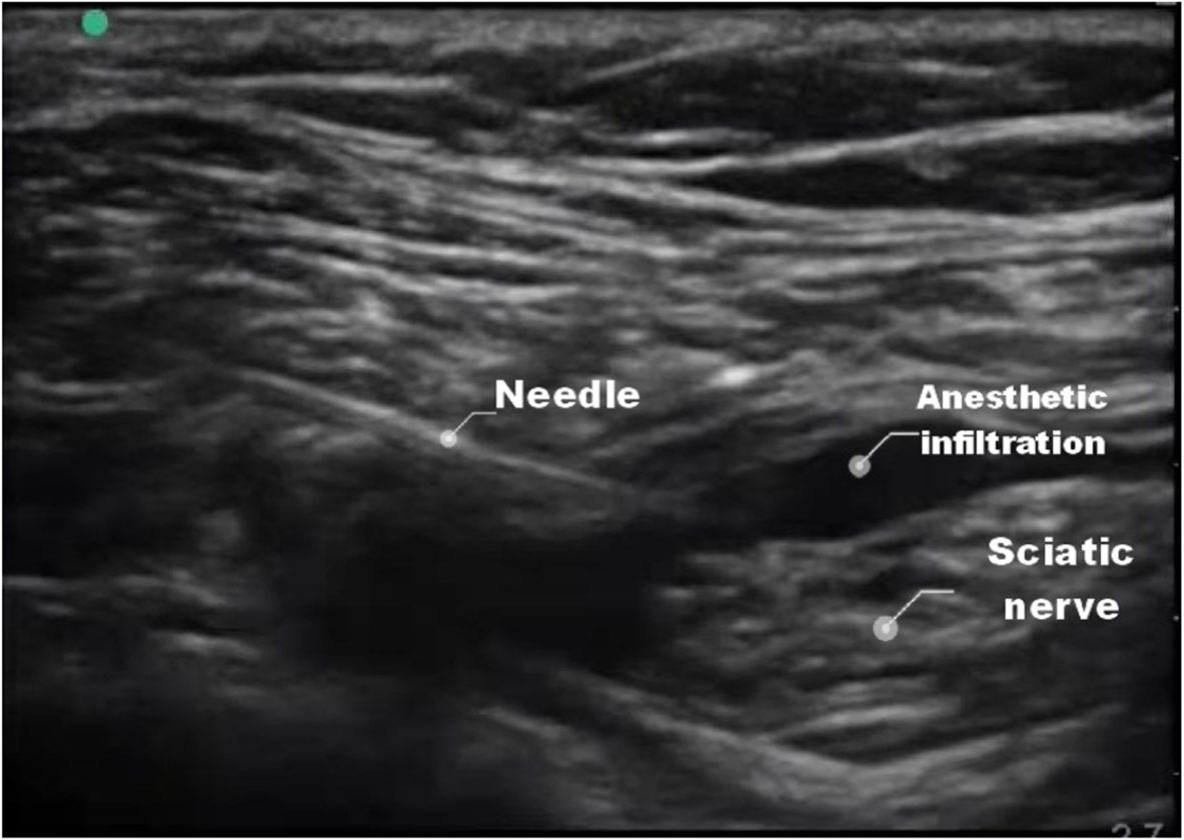
The landmarks of sciatic nerve block and local anesthetic injection under ultrasound view

After operation, the patients in group B were kept in supine position, and the affected limb was placed on a cushion and kept in flexion to facilitate the placement and operation of ultrasound probe. Subsequently, they received ultrasound-guided single popliteal sciatic nerve block. Terason 2000 ultrasound probe (frequency 5–10 MHz, Sonosite company, USA) was used to guide the localization and 100 mm nerve stimulation needle (B. Braun company, Germany, Batch number: 20E22H8B01) was used for nerve block. The nerve stimulation needle is only used for puncture, not connected to the nerve stimulator. After disinfection, a high-frequency linear probe was used to scan the popliteal fossa transversely, and the ultrasound beam was perpendicular to the sciatic nerve to identify separate tibial and common peroneal nerves. Movement of the probe proximally brought tibial and common peroneal nerves together to form the sciatic nerve at a variable point, above the popliteal crease. A 21-gauge, 100-mm insulated needle (20E22H8B01, Braun, Germany) was inserted using an in-plane technique, 20 ml of 0.5% ropivacaine (100 mg/10 ml, Batch number: H20020248, AstraZeneca, Sweden) was deposited on either side of the sciatic nerve after negative aspiration. During the injection process, the needle-tip position was adjusted as necessary to ensure circumferential spread of local anesthetic around the sciatic nerve (Fig. 1).

A patient-controlled intravenous analgesia (PCIA) pump (TUOREN, 1.5 μg/kg sufentanil and 6 mg tropisetron; flow rate: 2 mL/h; bolus: 0.5 mL; lockout time: 15 mins) was connected but switched off for patients in two groups. When they felt uncomfortable, they could use the analgesia pump independently. They could press PCIA pump when experiencing severe pain. If the patient asked for more painkillers, the pain management plan of the ward was intramuscular ketorolac 30 mg.

### Outcome measures

The time elapsed between discharge from operating room and initiation of analgesia pump (Ta), the time of first pressing the analgesia pump (Tb), duration of analgesia pump use and the total number of times the patient pressed the analgesia pump were recorded. The times of rescue analgesia and the adverse reactions caused by the analgesics such as pruritus, nausea and vomiting were recorded. The visual assessment scale (VAS) was used to assess the pain of patients immediately after discharge from the operating room (T1), at 4th (T2), 8th (T3), 12th (T4), 16th (T5), 24th (T6) and 48th (T7) hour after operation. The satisfaction of surgeon and nurse and the satisfaction of patients were collected respectively. The satisfaction of surgeon and nurse were assessed by questioning them whether their patients with calcaneal fracture would choose the same analgesic method in the future. If their answers were yes, it will be recorded as satisfaction. If their answers were different or no, it will be recorded as dissatisfaction. The satisfaction of patients were assessed by questioning them whether they would receive the same analgesic method in the future.

### Sample size estimation and statistical analyses

The sample size of the study was calculated using the G*Power program (V.3.1.9). We aimed to show a significant difference in VAS score after surgery. According to our preliminary experimental results, we estimate the mean VAS score (SD) is 5.1 in group A and 0.3 group B at 8 h after the surgery. The required sample size was thus 42 subjects per group with 80% power and a two-tailed α error of 5%. Considering a high incidence of dropout, we decided to include 60 patients in each group.

Statistical analyses were performed with IBM SPSS Statistics for Windows, version 26.0 (IBM, Armonk, NY, USA). Numerical data fitting normal distribution were expressed as mean ± SD, and numerical data not fitting normal distribution were expressed as median (minimum-maximum). Categorical data were expressed as percentage. Difference between mean or median values of the numerical data was assessed with Student t test or Mann-Whitney U test according to distribution of the data. Significance between categorical data was assessed either with Mann Whitney U test or with chi-squared test. A two-sided *p*-value < 0.05 was considered significant.

## Results

**Fig. 2:**
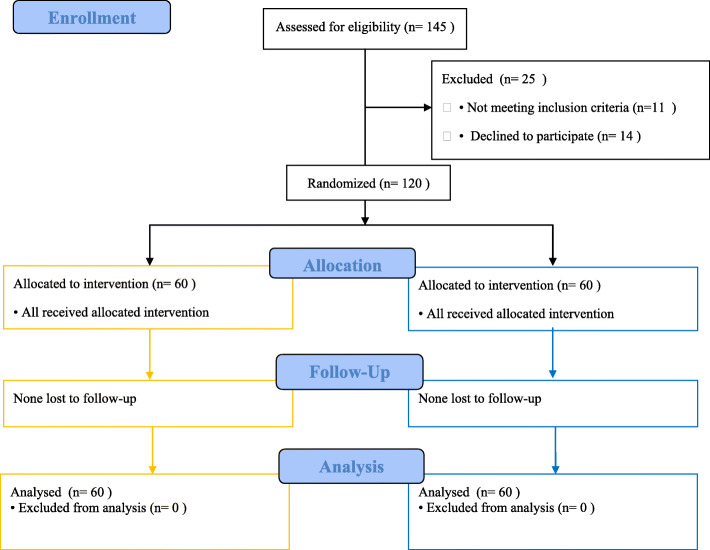
Flow diagram of study participants. Group A (Orange); Group B (Blue)

One hundred and forty five patients were assessed for study eligibility during this study. Eleven patients did not meet the inclusion criteria and 14 patients declined to participate in this study. Finally, 120 patients were randomized and completed the study (Fig. 2).

There were no statistically significant differences between the two groups with respect to age, weight, height, sex, ASA physical status, surgical site, surgical time, mechanism of injury, Sanders classification of calcaneal fractures. (Table 1).

**Table 1:** Demographic characteristics of the patients between two groups(*n* = 60, χ ± s)

	Group A	Group B	*P* value
Age (yrs)	46.25 ± 12.66	42.75 ± 12.76	0.349
Weight (kg)	68.67 ± 6.22	67.75 ± 4.78	0.813
Height (cm)	169.58 ± 5.67	170.23 ± 5.56	0.428
ASA physical status			0.552
I(%)	45 (75%)	47 (78.33%)	
II(%)	15 (25%)	13 (21.67%)	
Sex			0.476
Female(%)	43 (71.67%)	46 (76.67%)	
Male(%)	17 (28.33%)	14 (23.33%)	
Sanders Clicification			0.276
II	35	33	
III	25	27	
Mechanism of injury			0.662
Fall	43	45	
Traffic injury	9	8	
Other trauma	8	7	
Surgical site			0.386
Left foot(%)	38 (63.33%)	33 (55%)	
Right foot(%)	22 (36.67%)	27 (45%)	
Surgical time (min)	96.83 ± 12.07	94.17 ± 14.16	0.427

The Ta, Tb and the duration of analgesia pump use were lower in group A than in group B(*P* < 0.05). The total number of times the patient pressed the analgesia pump increased in group A than in group B(*P* < 0.05). Compared with group A, the times of postoperative rescue analgesia and adverse reaction related to analgesics such as nausea reduced in group B. But difference in vomiting and pruritus between two groups had no statistical difference (Table 2).

**Table 2:** Postoperative analgesia related data of the patients between two groups(*n* = 60, χ ± s)

	Group A	Group B	*P* value
Ta (h)	3.50 ± 0.47	13.45 ± 1.88	0.000
Tb(h)	4.28 ± 0.59	15.57 ± 2.99	0.000
Duration of analgesia pump use(h)	47.88 ± 0.27	49.55 ± 0.21	0.019
Number of times the patient pressed the analgesia pump	8.47 ± 1.11	1.82 ± 0.85	0.000
Rescue analgesia	0.15 ± 0.36	0.03 ± 0.18	0.000
Analgesics related adverse reactions			
Nausea	8 (11.09%)	2 (3.33%)	0.047
Vomiting	3 (5%)	0 (0%)	0.122
Pruritus	4 (6.67%)	1 (1.67%)	0.182

**Fig. 3:**
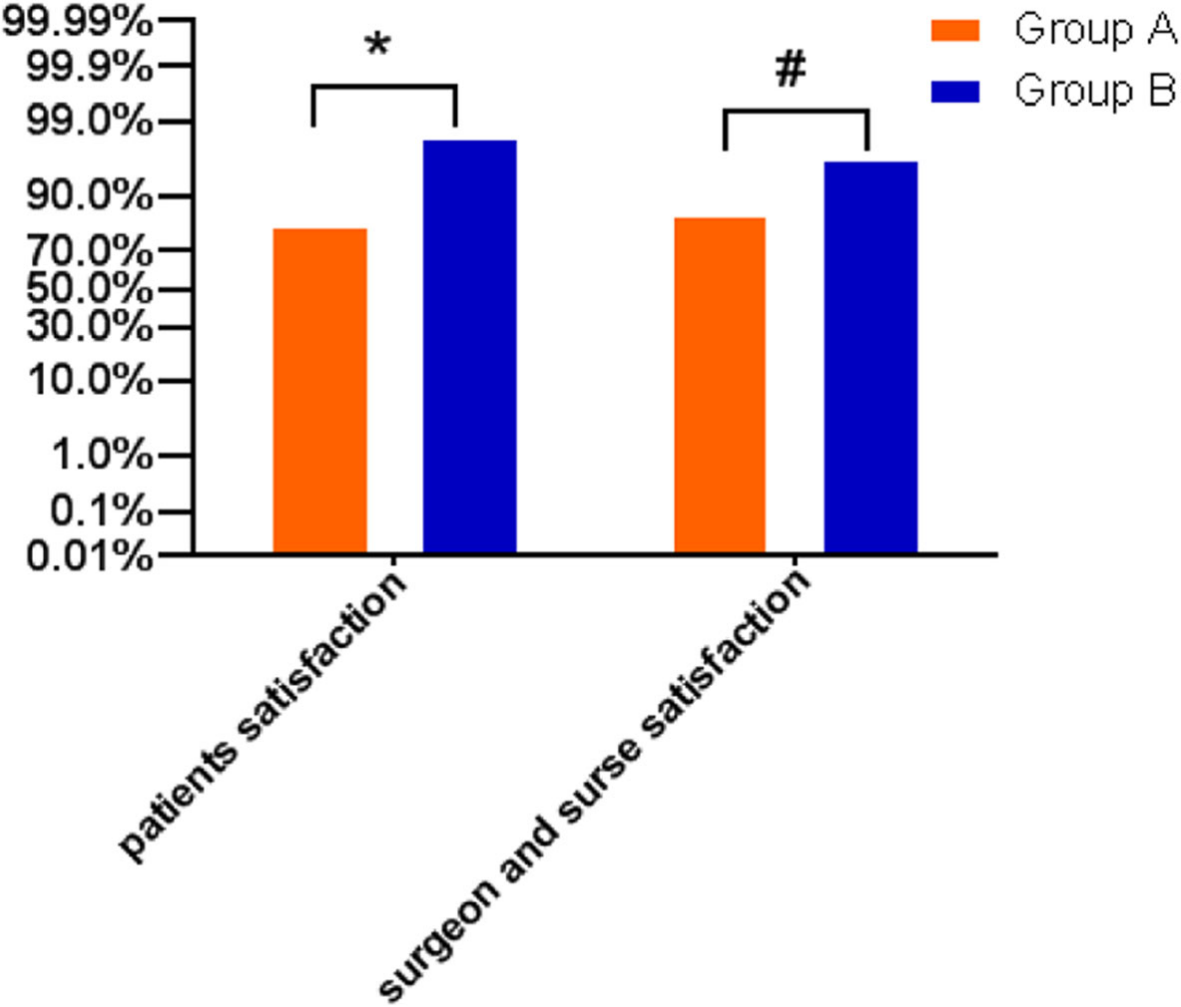
Patients, surgeon and nurse satisfaction between two groups. *P = 0.002; ^#^*P* = 0.027 Group A compared with Group B

Patient satisfaction [group A:81.67%, group B:98.33%; *P* = 0.002] and surgeon and nurse satisfaction [group A:85%, group B:96.67%; *P* = 0.027] was statistically higher in group B than in group A (Fig. 3).

**Fig. 4:**
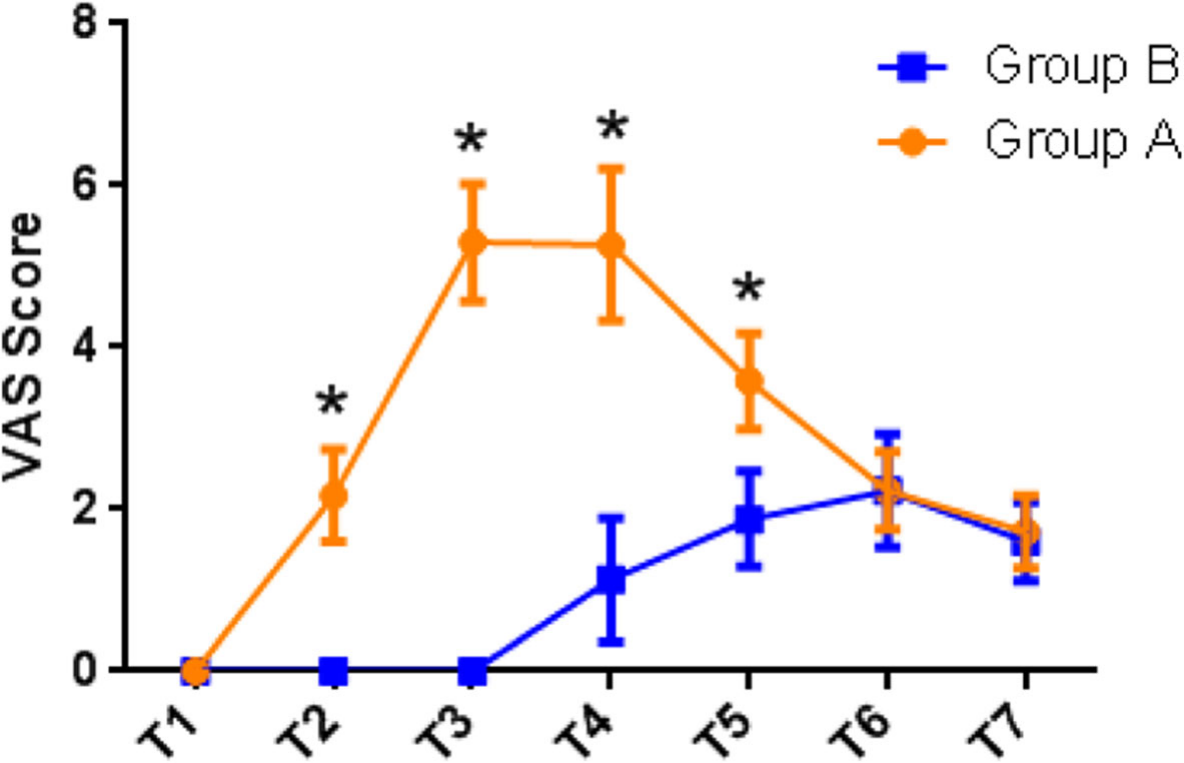
The VAS score at different time points between two groups. At T2, T3, T4, T5, *P<0.001 Group A compared with Group B

At T1, T6 and T7, no differences were found in VAS scores between the groups. From T2 to T5, pain scores were significantly lower in group B (T2: 0; T3: 0; T4: 1.13 ± 0.77; T5: 1.88 ± 1.19; *P* < 0.001) than in group A (T2: 2.1 ± 1.70; T3: 5.3 ± 0.72; T4: 5.27 ± 0.94; T5:3.58 ± 0.59; *P* < 0.001). (Fig. 4).

## Discussion

Foot surgery often causes serious and prolonged postoperative pain [19]. The primary aim of this study was to explore an effective method of analgesia for patients with unilateral calcaneal fractures. In this study, our results showed that implementing ultrasound-guided single popliteal sciatic nerve block in postoperative analgesia strategy for patients who received unilateral ORIF of calcaneal fracture could result in reduction of pain and analgesics consumption and improvement of satisfaction among patients significantly.

Calcaneal fracture is the most common tarsal fracture, accounting for about 60% of all tarsal fractures. 75% of calcaneus fractures are intra articular fractures, which is associated with high pain intensity after operation. With the disappearance of analgesic effect, patients begin to suffer from postoperative pain resulting from the surgical incision, experiencing the most severe pain at 24 h after operation [2, 7]. As most of the postoperative pain is acute nociceptive pain, and tissue injury may cause inflammatory reaction, the release of inflammatory and pain mediators reaches the peak within 24 h, aggravating ischemia, hypoxia and edema of the primary lesion [20]. In addition, postoperative pain can lead to hematoma, incision dehiscence and even infection, and patients are unable to walk normally, which can bring adverse postoperative experience for patients [21]. Therefore, it is very urgent to find an effective analgesic scheme for patients undergoing calcaneal fracture surgery. Effective analgesia can not only reduce postoperative pain, but also promote postoperative rehabilitation training and reduce postoperative complications.

Currently, there are a number of postoperative analgesia programs available for lower limb surgery, such as PCIA pump, continuous epidural analgesia, oral analgesics and peripheral nerve block. Opioids, such as sufentanil, are commonly used in PCIA pumps and oral analgesics [22]. It has been confirmed that opioid use is a high risk factor for postoperative nausea and vomiting (PONV) [10, 23]. PONV may not only cause blood pressure rise, wound tear, infection and other complications, but also lead to water electrolyte disorder and aspiration in severe cases, resulting in prolonged hospitalization and increased hospitalization expenses [24]. In this study, the incidence of nausea decreased statistically in group B. Although there were no significant differences in vomiting and pruritus, their incidence were still lower in group B than group A. Continuous epidural analgesia is also a common analgesic scheme. Anesthesiologists put the catheter into the patient’s epidural space and connect the PCIA pump, which is mostly used for postoperative analgesia in patients with lower limb surgery and labor analgesia [25]. Despite excellent analgesic properties, epidural analgesia has several disadvantages. Epidural analgesia is a kind of invasive procedure, which is more likely to cause infection at the puncture site. Because the fixation of epidural catheter is not perfect, there is a risk of catheter displacement, thereby affecting the process of rehabilitation training to a certain extent [26].

With the continuous development of ultrasound technology in the perioperative period, ultrasound-guided PNB has been widely used in the anesthesia and postoperative analgesia management of fracture surgery [12, 27]. The target of PNB in the treatment of pain is to block the transmission of nociceptive signal to the dorsal horn of spinal cord, therefore, no pain will be perceived. In the meantime, PNB can prevent the formation of synaptic long-term potentiation and central sensitization in the dorsal horn of spinal cord [28]. Ultrasound-guided PNB can reduce the probability of vascular puncture, the dosage of local anesthetics, the risk of systemic toxicity of local anesthetics, and the incidence of short-term recoverable nerve injury [29]. Cooper J have confirmed that single sciatic nerve block via popliteal fossa approach can be safely and effectively used in calcaneal fracture repair, providing patients with good intraoperative analgesia [30]. Meanwhile, several studies have reported faster analgesic effect of anesthesia drugs injected under the nerve sheath, lower dosage of drugs required, and better analgesic effect [31], however, other studies have suggested that the nerve injury is more likely to be caused by drugs injected under the nerve sheath [32]. In this study, we chose to inject anesthetics around the sciatic nerve and adjust the position of the needle to make the anesthetics fully infiltrate and surround the sciatic nerve. This method can provide a better blocking effect without damaging the sciatic nerve, and obtain higher success rate of nerve block.

Studies have found that pain intensity on the first day after surgery could be used to predict the recovery of patients [33]. Therefore, effective postoperative analgesia is very important, which can accelerate the recovery of patients. As the postoperative pain of calcaneal fracture is more severe, the conventional postoperative analgesia pump is difficult to meet the needs of patients. Therefore, they generally need to receive remedial analgesia. A large number of powerful opioids injected into patients in a short period of time are more likely to cause a series of complications, such as nausea and vomiting, respiratory depression, gastrointestinal function damage and so on. Some cases have been reported that patients with sciatic nerve block are prone to fulminant pain [34], therefore, we used sciatic nerve block combined with PCIA pump. Patients could press the analgesia pump when necessary. In this study, we found that the postoperative VAS score and the incidence of postoperative adverse reactions of patients in group B were significantly reduced, the time to initiation of analgesia was prolonged, and the number of PCIA compressions was increased, indicating that this analgesic scheme is safe and effective for patients undergoing calcaneal fracture repair.

Ropivacaine is a long-acting local anesthetic, which is advantageous in producing motor nerve separation, that is, exerting an analgesic effect without affecting the motor nerve of patients, therefore, it is preferred drug for nerve block. It has been proved that 0.5% ropivacaine can achieve satisfactory analgesic effect in sciatic nerve block, and there are no differences between 20, 30, or 40 mL 0.5% ropivacaine in continuous lateral popliteal sciatic-nerve block [35]. In order to reduce the local tissue damage caused by anesthetic dose and relieve the discomfort of patients caused by drug injection, we selected 20 mL 0.5% ropivacaine for ultrasound-guided sciatic nerve block for postoperative analgesia. The results of this study showed that the average time to initiation of analgesia in group B was 16 h, which was consistent with the results of the study by Fournier [35]. In addition, patients who received postoperative sciatic nerve block had higher satisfaction. The reason may be that the most severe period of postoperative pain was just within the action time of sciatic nerve block, and patients had good sleep quality on the night of operation and could get sufficient rest. In addition, the improvement of quality of life and the pleasure of the patients during the rehabilitation stage may be the reasons for improvement of satisfaction. The doctors and nurses in charge of these patients reported higher satisfaction for no additional postoperative analgesia is required.

It is undeniable that there are still some limitations in this study. In this study, only research indexes of patients within 2 days after operation were collected, frequency of assessment and the follow-up time still needs to be extended to obtain more accurate results.

## Conclusion

In conclusion, ultrasound-guided single popliteal sciatic nerve block can effectively reduce the postoperative pain of patients receiving unilateral ORIF of calcaneal fracture. In addition, it could reduce adverse reactions of analgesics and improve patient and surgeon satisfaction. Therefore, it can be safely and effectively applied to patients receiving unilateral ORIF of calcaneal fracture.

## Data Availability

The datasets generated during and/or analyzed during the current study will be available upon reasonable request from the third hospital of hebei medical university. Email: 18332336810@163.com
